# Intra-Brain Connectivity vs. Inter-Brain Connectivity in Gestures Reproduction: What Relationship?

**DOI:** 10.3390/brainsci11050577

**Published:** 2021-04-29

**Authors:** Michela Balconi, Giulia Fronda

**Affiliations:** 1 International Research Center for Cognitive Applied Neuroscience (IrcCAN), Catholic University of the Sacred Heart, 20122 Milan, Italy; michela.balconi@unicatt.it; 2 Research Unit in Affective and Social Neuroscience, Department of Psychology, Catholic University of the Sacred Heart, 20122 Milan, Italy

**Keywords:** gestures, EEG, intra-brain connectivity

## Abstract

Recently, the neurosciences have become interested in the investigation of neural responses associated with the use of gestures. This study focuses on the relationship between the intra-brain and inter-brain connectivity mechanisms underlying the execution of different categories of gestures (positive and negative affective, social, and informative) characterizing non-verbal interactions between thirteen couples of subjects, each composed of an encoder and a decoder. The study results underline a similar modulation of intra- and inter-brain connectivity for alpha, delta, and theta frequency bands in specific areas (frontal or posterior regions) depending on the type of gesture. Moreover, taking into account the gestures’ valence (positive or negative), a similar modulation of intra- and inter-brain connectivity in the left and right sides was observed. This study showed congruence in the intra-brain and inter-brain connectivity trend during the execution of different gestures, underlining how non-verbal exchanges might be characterized by intra-brain phase alignment and implicit mechanisms of mirroring and synchronization between the two individuals involved in the social exchange.

## 1. Introduction

Verbal and non-verbal communication processes are fundamental in individuals’ lives, characterizing their daily interactions and conveying information with different purposes [1]. Specifically, while words connote verbal interactions, non-verbal interactions are mediated by the use of gestures that can be considered facilitators of social interactions [2,3,4,5] via their influence on communicative meaning and interpersonal exchange dynamics [6].

In recent years, neuroscientific research has gradually moved towards understanding the neural mechanisms underlying the ability to observe, produce, recognize, and understand different types of gestures [7,8] with a different valence, which represents an essential aspect of human sociality [9]. This has led to the need to examine further how the affective connotations of non-verbal communication can influence non-verbal exchanges [10,11,12]. In terms of gestures’ complexity, some recent studies [13,14] have shown the involvement of important brain structures that compose the “human mirroring” system, such as the ventral and dorsal premotor cortex, the anterior inferior parietal lobule, the somatosensory areas, the middle temporal gyrus [15], and the frontal cortex [16]. Other studies have investigated the processes underlying the execution and observation of gestures, showing a direct link between action coding and decoding [17,18], which leads interagents involved in non-verbal interactions to perceive themselves as part of a proper joint action, thus developing “resonance mechanisms” and a “common perceptive base” [19,20]. Furthermore, the neural mechanisms—especially sensorimotor cortex responses—associated with the production of gestures have been investigated via electroencephalography (EEG), which has allowed for the detection of cortical oscillations that provided valuable information on transient local functional networks underlying gesture execution [21,22,23].

As observed in some EEG studies, high-frequency bands are involved in action perception [24,25] as well as gesture observation and execution [26,27,28], while low-frequency bands are involved in emotional processes modulating the production of own and other actions [29]. In the present study, an EEG hyperscanning approach was used to investigate the neural correlates underlying non-verbal communication exchanges connoted by different gestures.

Specifically, EEG in hyperscanning allows for a better temporal resolution in recording the two interagents’ interactions moment by moment [20,30]. Social communication is a complex phenomenon that cannot be fully traced back to the study of a single isolated brain [30,31,32], which was one of the limitations of some social cognition studies that have investigated social behavior off-line without considering individuals’ interactions and face-to-face exchanges [30,32,33,34].

The assumption of a two-person perspective, and the simultaneous recording of two participants’ brain activity using EEG in hyperscanning, allow researchers to examine hyperbrain functional networks, which include functional connectivity and allow for obtaining information about mechanisms of intra-brain and inter-brain connectivity [35]. As such, in this paper, individuals’ intra-brain connectivity patterns were calculated during the execution of different gestures conveying emotional, social, and informative contents [36,37,38,39]. Specifically, the present study aimed to investigate the relationship between intra-brain and inter-brain connectivity in dyads of interacting individuals (an encoder, who executed the gesture, and a decoder, who received the gesture) during the execution of different types of gestures (affective, social, and informative ones), both positively and negatively connoted. In particular, we wanted to investigate whether specific differences or similarities characterize the relationship between these two forms of connectivity (namely, intra-brain and inter-brain), and whether those two forms of connectivity influence each other, thus providing information about individuals’ functional connectivity mechanisms. Inter-brain connectivity is understood as the calculation of functional or effective connectivity between the brains of individuals involved in a social exchange [30], or during a social interaction [40]. Indeed, it has been observed that the behavior of two individuals involved in an interaction can spontaneously coordinate and lead to inter-personal coupling mechanisms, as occurring during verbal and non-verbal communication exchanges [36,41]. Specifically, the inter-brain connectivity mechanisms underlying non-verbal communication characterized by the use of affective, social, and informative gestures with a positive and negative valence have been investigated in the previous study by Balconi and Fronda [36], which highlighted the increase in inter-brain connectivity for high- and low-frequency bands in frontal and posterior regions, depending on the type of gesture that was executed. Moreover, a lateralized frontal inter-brain connectivity for high- and low-frequency bands was observed depending on the valence of the gestures. These mechanisms were observed in both subjects involved in the exchange (i.e., the encoder and the decoder) regardless of their interaction role. Unlike inter-brain connectivity, the intra-brain one refers to the coordinated neural activity or neural coupling between individuals’ brain regions. In particular, intra-brain connectivity provides information about the functional specialization of individuals’ brains and their representations about themselves and others [42,43].

Considering, therefore, the characteristics of those two forms of connectivity and based on the evidence that emerged in the previous study by Balconi and Fronda [36], the present study aimed to observe whether intra-brain connectivity follows the same pattern of modulations observed for the inter-brain one, and if inter-brain synchronization is related to the anatomical and functional similarity of the two individual brains in interaction. In light of this aim, based on previous results related to the role of gesture types and inter-brain measures [36], we expected to observe a decrease in alpha and an increase in delta and theta intra-brain connectivity in the frontal region, compared with the other ones, for affective and social gestures. Considering the meaning of such gestures, the frontal regions might act as mediators of the emotional mechanisms implicated in socio-emotional processes and emotional intelligence [44,45,46,47,48,49]. Furthermore, we expected to find a decrease in alpha intra-brain connectivity in the posterior region (temporo-parietal) for informative gestures, due to the implication of attentional mechanisms [50,51].

Furthermore, focusing on gestures’ valence, we expected to observe an increase in intra-brain connectivity in the left frontal areas for positive gestures, which likely induce an “approaching drive”, compared to negatively valenced gestures, which likely induce an “avoidance drive” [52].

Finally, focusing on the role of interagents, we expected to observe a similar responsiveness in both the encoder and decoder due to the mechanisms of implicit coupling and synchrony that occur during gestural communication. As shown by previous studies [19,53], behavioral coordination and synthonization between interagents increase during joint actions, such as non-verbal exchanges, and foster inter-brain connectivity between individuals [36,37,54]. In light of that evidence, we hypothesized that we would observe an increase in delta and theta intra-brain connectivity in the frontal area for affective and social gestures, and in the parietal area for informative ones, in both the encoder and the decoder, due to mirroring and resonance mechanisms experienced during gestural communication. 

## 2. Materials and Methods

### 2.1. Sample

Thirteen couples of university students (M_age_ = 23.05; SD_age_ = 2.78) were recruited. Eleven dyads of subjects were the same as in the previous work of Balconi and Fronda [36], but the analyses reported here have not been previously published. Participants were paired in dyads composed of individuals of the same gender who were not familiar with each other and who were assigned the role of encoder or decoder.

Participants had normal or corrected-to-normal vision and they were all right-handed. The following exclusion criteria were used for the participants’ recruitment: age <18 and >40; clinical history of psychiatric or neurological disorders; presence of cognitive or neurological deficits; clinically relevant stress level, measured with the use of the perceived stress scale (PSS); and experience of significant stressful life events in the last 6 months. The subjects received no payment for their participation, and gave their informed consent to participate in the research. The study followed the principles and guidelines of the Helsinki Declaration and was approved by the local ethics committee of the Department of Psychology of the Catholic University of the Sacred Heart and by the University of Lille committee.

### 2.2. Procedure

**Figure 1:**
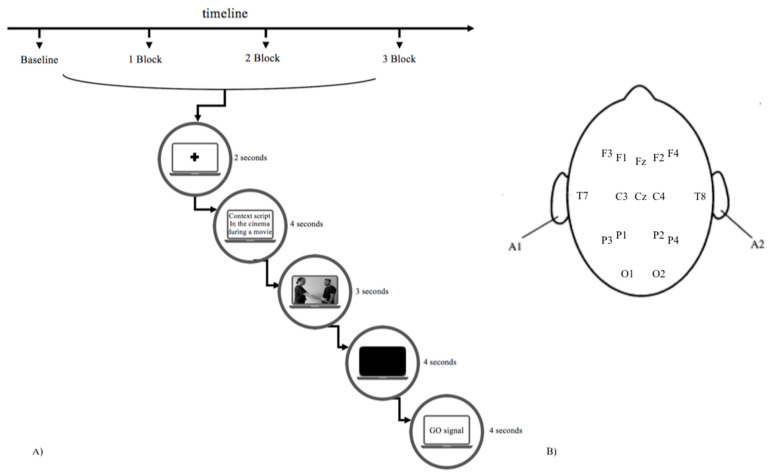
(**A**) Experimental procedure for the task administration. The figure shows the trial structure used in the three task blocks. (**B**) The figure shows the location of EEG channels. The electrodes were located in the following positions: F3, F1, Fz, F2, F4, T7, C3, Cz, C4, T8, P3, P1, P2, P4, O1, and O2.

Participants were asked to sit one in front of the other at a distance of 60 cm from a computer placed in a room. In particular, participants were involved in a task, administered through the E-Prime 2.0 software (Psychology Software Tools Inc., Sharpsburg, PA, USA), according to which they watched short videos reproducing a gestural communication between two individuals and then re-enacted it. Namely, for each dyad, one individual (the encoder) was asked to observe and reproduce the gesture seen in the video toward his/her partner (the decoder), who was, instead, asked to observe the reproduced gesture. The task presentation was divided into three blocks, each composed of randomized stimuli, with the following structure: an initial blank screen (2-second duration), a short description of the context of observed interactions (4-second duration), the gesture presentation video (3-second duration), a blank inter-stimulus (4-second duration), and a final “go” signal to reproduce the gesture (Figure 1A) [36,37,38,39]. The videos presented gestures of different types (namely, social, affective, and informative ones), and of different valence (namely, positive and negative). For each gesture, 10 videos were presented. Half of the videos (*n* = 30) depicted a woman–woman interaction, the remaining half depicted a man–man interaction. As an example of the gesture type and valence pairing, the videos of social positive gestures reproduced gestures that were meant to start or establish a relationship with another individual, while the social negative ones reproduced gestures aimed at finishing or interrupting an interpersonal relationship.

The affective positive videos reproduced gestures that aimed to show a state of physical and psychological well-being to the interlocutor, while affective negative ones aimed to convey a state of psychological and physical discomfort. Finally, the videos reproducing informative gestures aimed to describe an informative situation without social or emotional content. The positive or negative valence for informative gestures was attributed to the context shown before the video [36,37,38,39].

The implementation of the task was preceded by a preliminary phase dedicated to stimuli validation, which involved 14 judges (M_age_ = 28.34, SD_age_ = 0.04) who were asked to evaluate, using a seven-point Likert scale, the emotional impact, familiarity, social meaning, concreteness, complexity, commonality, and use frequency of each gesture. The definition of stimuli categories and their primary features were tested via statistical analysis [36,37,38,39].

### 2.3. EEG Recording and Analysis

In order to record EEG activity, two EEG systems with a 16-channel montage were employed (V-Amp and LiveAmp; Brain Products GmbH, Gliching, Germany). The electrodes were placed, via two ElectroCaps, in correspondence to the following sites: F3, F1, Fz, F2, F4, T7, T8, C3, Cz, C4, P3, P1, P2, P4, O1, and O2. To detect eye movements, an EOG electrode was placed on the external corner of the eye (Figure 1B) [36,37]. Data were sampled at 1000 Hz. A 0.01–200 Hz input filter was used together with a 50 Hz notch filter. During data acquisition, electrode impedance was kept below 5 kΩ. Data were then filtered offline before processing by using a 0.5–40 Hz bandpass filter. The standard offline average reference was calculated to reduce signal–noise ratio. A geometric correction scheme (GSC) [55] was applied to suppress spatial leakage emanating from the seed location.

During the offline signal processing, data that included ocular and motor artifacts were excluded from the following steps. The EEG traces were segmented concerning the various conditions and, for each of these, the segments containing residual motor, ocular, or muscle artifacts were eliminated through manual artifact rejection by an expert in biological EEG signals. After this step, only the segments without artifacts were considered for the next steps.

Finally, power spectra were calculated to extract information on high- and low-frequency EEG bands: delta (0.5–4 Hz), theta (4–8 Hz), alpha (8–12 Hz), beta (14–20 Hz) [36,37,40]. In particular, the mean EEG power was extracted for each channel, each frequency band, and each condition to explore the neural correlates underlying gesture execution. Only the gesture execution phases were averaged, using a 4-second time interval.

### 2.4. Data Analysis

The intra-brain connectivity was calculated for each EEG band.

Specifically, the intra-brain connectivity was computed using the same algorithm of inter-brain connectivity [36,37] referring to two distinct matrices of data. In particular, the partial correlation coefficient Π_ij_ was computed (Equation (2)). It was obtained by normalizing the inverse of the covariance matrix Γ = Σ ^−1^: (Equation (1))

Γ = (Γ_ij_) = Σ ^−1^ inverse of the covariance matrix (1)

Π_ij_ = (−Γ_ij_)/√Γ_ii_Γ_jj_ partial correlation matrix (2)

Then, an ANOVA was applied to the intra-brain measures. The focus on intra-brain connectivity was to observe whether the execution of different gestures also produced consistent patterns of connectivity between individuals’ brain areas, and to compare these observations to those concerning inter-brain synthonization investigated in the previous study by Balconi and Fronda [36].

The ANOVA models included, as independent measures: role (encoder/decoder, 2), valence (positive/negative, 2), lateralization (left/right, 2), gesture (social/affective/informative, 3), and ROI (four regions of interest). Different ROI were computed for left/right analogous sites based on the following channels: frontal (F3, F1-F2, F4), central (C3, C4), temporo-parietal (T7, P1-T8, P2), and occipital (O1, O2) [36,37]. 

Greenhouse–Geisser epsilon was used to correct degrees of freedom in the ANOVA models when needed. Moreover, a Bonferroni correction for multiple comparisons was used in post-hoc comparisons. The normality of the data distribution was previously verified by checking kurtosis and asymmetry measures.

## 3. Results

### 3.1. Alpha Band

**Figure 2:**
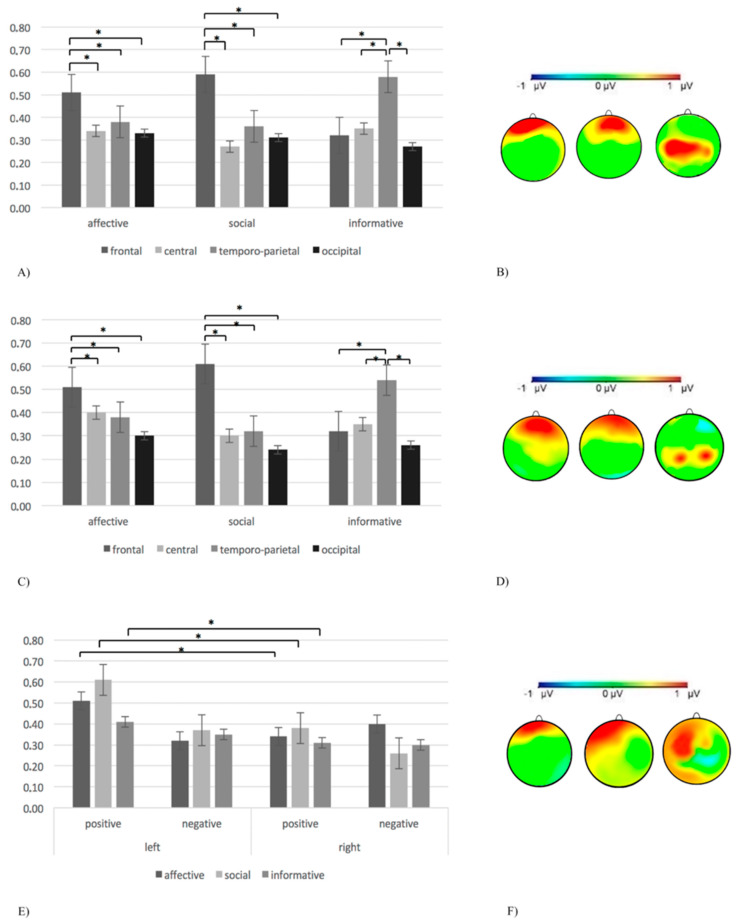
(**A**) A bar chart of alpha intra-brain connectivity for the three types of gestures (affective, social, and informative) in the frontal, central, temporo-parietal, and occipital areas. The bar chart shows the increase in frontal alpha intra-brain connectivity for affective and social gestures, and the increase in posterior alpha intra-brain connectivity for informative ones. Bars indicate ±1 SE. Statistically significant pairwise comparisons (*p* < 0.05) are marked by an asterisk. (**B**) Alpha intra-brain connectivity representations for affective (left side), social (middle), and informative (right side) gestures. The increase in alpha intra-brain connectivity is marked by the red area. (**C**) A bar chart of delta intra-brain connectivity for the three types of gestures (affective, social, and informative) in frontal, central, temporo-parietal, and occipital areas. The bar chart shows the increase in frontal delta intra-brain connectivity for the affective and social gestures, and the increase in posterior delta intra-brain connectivity for the informative gestures. Bars indicate ±1 SE. Statistically significant pairwise comparisons (*p* < 0.05) are marked by an asterisk. (**D**) Delta intra-brain connectivity representations for affective (left side), social (middle), and informative (right side) gestures. The increase in delta intra-brain connectivity is marked by the red area. (**E**) A bar chart of theta intra-brain connectivity for affective, social, and informative positive and negative gestures in the left and right cerebral side. The bar chart shows the increase in left frontal theta intra-brain connectivity for positive gestures. Bars indicate ±1 SE. Statistically significant pairwise comparisons (*p* < 0.05) are marked by an asterisk. (**F**) Theta intra-brain connectivity representations for affective (left side), social (middle), and informative (right side) gestures. The increase in theta intra-brain connectivity is marked by the red area.

Concerning alpha intra-brain connectivity, the ANOVA model showed a significant gesture x ROI interaction effect (F(6,154) = 10.11; *p* < 0.001; η^2^ = 0.37). Post-hoc comparisons highlighted higher alpha intra-brain connectivity in frontal areas compared to other ROIs for affective and social gestures, and in posterior areas compared to other ROIs for informative ones (all post-hoc comparisons *p* ≤ 0.001) (Figure 2A,B, Table 1). 

### 3.2. Delta Band

Concerning delta intra-brain connectivity, the ANOVA model showed a significant gesture x ROI interaction effect (F(6,154) = 9.12; *p* < 0.001; η^2^ = 0.35). In particular, post-hoc comparisons showed higher delta intra-brain connectivity in the frontal region compared to other ROIs for the affective and social gestures, and in the posterior (temporo-parietal) region for the informative ones (all post-hoc comparisons *p* ≤ 0.001) (Figure 2C,D, Table 1).

### 3.3. Theta Band

Concerning the theta intra-brain connectivity, the ANOVA model showed a significant valence x lateralization x gesture x ROI interaction effect (F(6,154) = 10.78; *p* < 0.001; η^2^ = 0.37). In particular, higher theta intra-brain connectivity in the frontal region with respect to other ROIs was highlighted by post-hoc comparisons for affective and social gestures (all post-hoc comparisons *p* ≤ 0.001). Furthermore, an increase in theta intra-brain connectivity for positive gestures was revealed in the left side as compared to the right one (F[1,25] = 9.77; *p* < 0.001; η^2^ = 0.37) (Figure 2E,F, Table 1).

### 3.4. Beta Band

Concerning the beta band, the ANOVA model did not show significant effects.

## 4. Discussion

The present study investigated the brain mechanisms involved in reproducing different categories of gestures with different valences during a real gestural interaction between an encoder and a decoder. In particular, the computation of intra-brain connectivity was used to explore intrapersonal cortical synchronization mechanisms. Furthermore, the present study aimed to explore the relationship between inter-brain connectivity mechanisms, investigated in the previous research by Balconi and Fronda [36], and intra-brain ones, during a non-verbal communication exchange characterized by affective, social, and informative gestures with positive and negative valences. While inter-brain connectivity provides information on cerebral connectivity patterns related to the relationship between the two interagents’ brains, the intra-brain one provides information on the relationship between the activity of different regions within each interagent’s brain, depending on the different types and valence of gestures. Therefore, comparing those two forms of connectivity is important because it allows us to understand the relationship between “individuals’ brains” and “interactional brains”, and allows us to understand the influence that those forms of connectivity can have on each other.

In particular, taking into consideration the present study’s results concerning the gestures type (affective, social, and informative), the intra-brain connectivity analysis highlighted an increase in alpha, delta, and theta intra-brain connectivity in frontal areas associated with social and affective gestures. Moreover, an increase in alpha and delta intra-brain connectivity was also observed in temporo-parietal areas in association with informative gestures. That different increases in the intra-brain connectivity depend on the gesture category might hint at the mirroring mechanisms of some cerebral regions that appear to be involved in action observation and execution [56,57,58,59,60]. The frontal and posterior regions appear to be involved in the mirroring mechanisms activated during action execution and the observation of the same action, creating an interdependent connection between encoder and decoder [17,18].

In comparing the present results to the inter-brain connectivity modulations observed in the previous work by Balconi and Fronda [36], a similar trend in association to gesture type emerged. In inter-brain analyses, an increase in alpha, delta, and theta inter-brain connectivity was observed in the frontal areas during the enaction of affective and social gestures, and in the posterior areas during the enaction of informative gestures.

The comparison of the two forms of connectivity (intra- and inter-brain connectivity) according to the type of gesture demonstrated that during the exchange of affective, social, and informative gestures, in addition to inter-brain connectivity mechanisms, intra-brain ones also occurred in specific brain regions. This highlights that, during the non-verbal exchange, an inter-individual synchronization occurs, which is, at the same time, related to the anatomical and functional similarity of the two interacting individuals’ single brains. This increase in intra-brain connectivity in the frontal and posterior areas (depending on the gesture category) was observed both for the encoder and the decoder, underlining similar internal connectivity [17,18]. A similar pattern was also observed for inter-brain connectivity, highlighting how the individuals involved in the exchange developed implicit coupling mechanisms due to greater emotional and attentional engagement during the interaction. This similar trend of inter- and intra-brain connectivity could be due to the fact that the increase in frontal and posterior inter-brain connectivity for the low-frequency bands (which are more involved in emotional processing [36,44,48,61,62]) and high-frequency bands (which are more involved in perceptual/attentional processes [36,50,51]) has led to an increase in interagent individuals’ emotional and attentive synchronization, which might have facilitated a consequent increase in intra-brain connectivity in single interacting individuals’ specific brain regions. Previous studies have also observed that an increase in intra-brain connectivity is strongly related to the level of cerebral and behavioral synchronization that develops between the two individuals involved in the exchange during the interaction [1,19].

Moreover, taking into consideration intra-brain connectivity depending on gestures’ valence, an increase in theta intra-brain connectivity has emerged in the left frontal areas for positive gestures compared to negative ones, emphasizing the role of the theta band in emotional processes [63,64,65,66].

As for the gestures’ type, the presence of intra-brain connectivity in the encoder and decoder has also been observed as related to gesture valence, as a marker of underlying resonance mechanisms [19,20] and implicit coupling [42]. Comparing this result with what has emerged for inter-brain connectivity in the previous study by Balconi and Fronda [36], a similar trend between the two forms of connectivity has been observed only for the theta band during the reproduction of positive gestures. In fact, in the previous study by Balconi and Fronda, an increase in inter-brain connectivity for both the theta and the beta band has emerged to a greater extent on the left side compared to the right one during the reproduction of positive gestures. In the case of the present study, however, the increase in intra-brain connectivity in the left hemisphere according to positive gestures’ valence occurred instead only for the theta band, consistently with the involvement of this frequency band in visual emotional stimulation [65]. In particular, it was observed how this frequency band responds to the emotional connotation of stimuli and the valence of emotional affective processes [61], which would involve both the encoder and the decoder.

Finally, no difference has emerged depending on the role of interacting individuals (i.e., encoder or decoder). A similar trend in the two forms of connectivity (intra- and inter-brain) also emerged, supporting the previous work by Balconi and Fronda [36], and highlighting the presence of some mirroring and implicit coupling mechanisms during the non-verbal interaction in both individuals involved in the exchange (encoder and decoder).

## 5. Conclusions

To conclude, the present results highlight the presence of common modulation mechanisms between intra- and inter-brain connectivity during the non-verbal exchange, depending on the category and valence of the reproduced gestures. From this evidence, and the comparison with what had been previously observed in the work by Balconi and Fronda [36], a relationship emerges between those two forms of connectivity, within which it appears that one facilitates the other. Indeed, in the present study, the intra-brain connectivity findings show how, regardless of the interagents’ role, an increase in connectivity occurs in the same brain areas depending on the gesture’s category and valence. This result supports the findings of the previous study by Balconi and Fronda [36] concerning the mechanisms of inter-brain connectivity, in which the presence of implicit coupling mechanisms in both subjects according to the type and valence of the reproduced gestures was observed. Indeed, it has been observed that the presence of coupling mechanisms between individuals involved in the exchange, which facilitate the formation of implicit sensorimotor mechanisms, are related to the anatomical connectivity mechanisms of single areas of the human brain. This, therefore, demonstrates a mutual influence of those two forms of connectivity, in which one seems to facilitate the other and vice versa.

On the one hand, as already demonstrated by previous studies [19,42,66], the presence of mechanisms of functional–anatomical similarity between the individuals involved in an exchange facilitates the creation of a sensory–motor coupling between interacting individuals. On the other hand, those sensorimotor coupling mechanisms, leading to an increase in interagents’ emotional, attentive, and behavioral synthonization, facilitates a consequent increase in intra-brain connectivity in the single brains of individuals involved in the exchange. Despite its originality, the present study is not without limitations, such as the fact that an increase in sample size could improve the generalizability of the results. Furthermore, integrating data collection with neuroimaging or peripheral measures could strengthen the empirical observations and enrich data interpretation, providing new evidence about the neural mechanisms underlying gestures’ reproduction. Another limitation of the research may be related to the limited number of channels used to record cortical activity. In this regard, future implementations of this research should consider different re-referencing procedures (e.g., rREST [67]) and the acquisition of the EEG signal considering a more significant number of channels. However, this problem could be partially reduced by the statistical approach, which considers the correlation values rather than simple values, eliminating the limitation related to the reduced number of channels. Finally, future studies, to overcome a last possible limitation of this study, could consider expanding the correlational analysis methodology underlying the calculation of intra-brain connectivity.

## Figures and Tables

**Table 1:** Table of intra-brain connectivity results (means of intra-brain connectivity).

**Frequency Bands**	**Gesture x ROI**											
	**Affective**				**Social**				**Informative**			
	**Frontal**	**Central**	**Temporo-Parietal**	**Occipital**	**Frontal**	**Central**	**Temporo-Parietal**	**Occipital**	**Frontal**	**Central**	**Temporo-Parietal**	**Occipital**
Alpha band	0.51	0.34	0.38	0.33	0.59	0.27	0.36	0.31	0.32	0.35	0.58	0.27
Delta band	0.51	0.40	0.38	0.30	0.61	0.30	0.32	0.24	0.32	0.35	0.54	0.26
	**Valence x Lateralization x Gesture x ROI**											
	**frontal**											
	**affective**				**social**				**informative**			
	**positive**		**negative**		**positive**		**negative**		**positive**		**negative**	
	**left**	**right**	**left**	**right**	**left**	**right**	**left**	**right**	**left**	**right**	**left**	**right**
Theta band	0.51	0.34	0.32	0.40	0.61	0.38	0.37	0.26	0.41	0.31	0.35	0.30

## Data Availability

The data that support the findings of this study are available from the corresponding author, [GF], upon reasonable request.
